# Exploring Nutrient-Adequate Sustainable Diet Scenarios That Are Plant-Based but Animal-Optimized

**DOI:** 10.3390/nu17020343

**Published:** 2025-01-18

**Authors:** Thom Huppertz, Luuk Blom, Lionel van Est, Stephan Peters

**Affiliations:** 1 School of Food and Nutritional Sciences, University College Cork, T12 Y337 Cork, Ireland; 2 Food Quality & Design Group, Wageningen University & Research, 6708 PB Wageningen, The Netherlands; 3 FrieslandCampina, 3818 LE Amersfoort, The Netherlands; 4 Nutrisoft, 9471 JE Groningen, The Netherlands; 5 Nederlandse Zuivel Organisatie (NZO), 2596 BC The Hague, The Netherlands; peters@nzo.nl

**Keywords:** diet, optimization, sustainability, linear programming, environmental impact

## Abstract

**Background**: Transitions toward more sustainable food systems may become rather polarized, particularly in the plant-based vs. animal-based debate. These discussions, however, are often based on environmental impact data from individual products or product groups and do not consider that the products together should form a nutrient-adequate diet that is also affordable. Linear programming approaches are ideal for exploring the impact of changes in diet composition on environmental impact and price while ensuring nutrient adequacy. **Methods**: In this study, we used a linear programming system, Optimeal 2.0, based on the diet in the Netherlands to explore the impact of changing the contributions of specific food groups on the environmental impact and price of the diet. **Results**: Reducing the amount of meat products in the diet reduced the environmental impact but increased the price. On the other hand, when dairy products were reduced or even omitted, the environmental impact of the nutrient-adequate optimized diet did not change notably, but prices increased notably. This could be attributed to the fact that the products required to compensate for the nutrient gaps by removing dairy products have equal environmental impact and higher prices. Increasing vegetable or fruit consumption increased price but did not affect the environmental impact nor did it increase the consumption of beans and pulses when kept within realistic levels. **Conclusions**: Overall, this work strongly highlights that consideration of ‘sustainability’ at a food product level is insufficient and that their role in nutrient-adequate diets needs to be considered. Furthermore, dietary shifts should be considered from the perspective of affordability and consumer preferences.

## 1. Introduction

Global food systems are under increasing pressure to deliver healthy and nutritious diets within planetary boundaries [1]. The environmental impact of current food production is higher than is acceptable for planetary health. On the one hand, this necessitates measures to reduce the impact of current food production. On the other hand, this also warrants dietary changes [1]. Particular emphasis has been paid in this respect to animal-based food products, such as meat and dairy products, whose environmental impact on a mass or volume basis is higher than that of many plant-based food products [2]. However, it has also been argued that some animal-based products bring a unique set of nutrients to the diet, which is not readily replaced by plant-based products [3,4,5]. Furthermore, looking beyond solely nutrients, diets low in milk have been classified as a dietary risk factor in the global burden of disease (GBD) studies due to the increased risk of colorectal cancer at low milk intake [6]. It is, thus, critical to consider not only the environmental impact of food products but also their nutritional value.

The environmental impact of food products is most often assessed by the so-called life cycle assessment (LCA), which can be described as a systematic analysis of the environmental impact of a product over its entire life cycle [7]. This includes, e.g., material and energy requirements throughout the supply chain of a product. A typical cradle-to-grave LCA includes raw materials, product manufacture (including packaging), distribution, and use as well as recycling or disposal of materials [8]. While global warming potential (GWP), expressed in CO_2_ equivalents (CO_2_-eq), is the most often discussed unit of environmental impact, LCA analysis can also include other environmental impact parameters, such as land use, freshwater eutrophication, fossil resource scarcity, marine eutrophication, water consumption, and terrestrial acidification [8,9]. Functional units of LCA are typically mass- or volume-based, e.g., kg CO_2_-eq per kg of product [7,8]. Aspects that are not included in the (standard) LCA of food products include nutritional value and price. While they are indeed not required for the primary purpose of an LCA, i.e., assessing environmental impact, they are important for putting the outcomes of LCAs into perspective when it comes to dietary changes.

The inclusion of the nutritional value of food products in LCA is addressed in a so-called nutritional LCA (nLCA). In an nLCA, a (complimentary) functional unit is not mass- or volume-based but becomes nutrition-based [9]. Various options exist for nutrition-based functional units in nLCA, such as those based on the energy content or on single nutrients, which may also include qualitative aspects, such as protein quality [10,11,12]. However, more balanced assessments in nLCA are typically achieved when using nutrient profiling scores, such as the nutrient-rich foods (NRF) index or other nutrient profiling scores [10]. While nLCA outcomes allow a more balanced comparison of food items by also including nutritional aspects, considerable challenges remain. Arguably, the biggest one is that food products are part of a diet and that nutritional adequacy can only be assessed on a dietary basis. Hence, considering or comparing single products only provides limited insights, if the dietary context is not considered. This is, for instance, important when considering the importance of specific nutrient contributions. While some nutrient requirements are readily met in a dietary context, others, such as e.g., Ca, may be far more challenging [13]. Hence, ideally, diet-based approaches are used.

While product-based nLCA approaches are relatively easy from a computational perspective, diet-based approaches, however, can provide some additional challenges, particularly around data availability. While the nutrient content and composition of food products are often readily available from (national) food composition databases, LCA data on products are scarcer and often do not cover an entire diet. However, there are good examples where complete datasets have allowed diet optimization in the sustainability context without compromising nutritional adequacy. One such model, also used in this study, is the Optimeal 2.0 model, which is based on linear or quadratic programming and was developed from a Dutch perspective and was previously applied to explore the influence of dietary changes in the context of reducing environmental impact [14,15,16,17,18]. While such studies do provide insight into what a future diet could look like, they typically do not result in immediate dietary changes because gaps between current and new diets may be too large to enable consumer change. However, instead of setting specific targets for environmental impact indicators, such as GWP or land use, as performed in previous studies [14,15,16,17,18], these models can also be used to investigate the influence of shifts in dietary patterns by increasing or decreasing the intake levels of certain food groups, on dietary shifts as well as associated environmental impact. In the current study, we undertook such investigation, whereby intake levels of one food group were fixed and the impact on diet composition and environmental impact was assessed. In addition, product price was also included as an outcome. In the current study, particular emphasis was placed on the food groups meat, dairy, vegetables, fruits and beans and pulses. In addition to looking at diet composition, environmental impact, and price, we also investigated nutrient profiles of optimized diets, with the aim of identifying which nutrients are critical drivers in sustainable diet optimization.

## 2. Materials and Methods

### 2.1. Experimental Setup

In this study, we used the Optimeal^®^ 2.0 (Mérieux NutriSciences | Blonk, Gouda, The Netherlands) modeling platform previously described [14,17] to investigate the impact of dietary shifts in the Netherlands. Optimeal^®^ 2.0 can propose dietary shifts from the current diet based on nutritional or environmental dietary goals, optimizing for popularity, by searching for scenarios of foods that resemble current diets as closely as possible. The starting point for optimization was a diet representative of a typical Dutch diet based on the Dutch National Food Consumption Survey, as described in detail by Kramer et al. [14] and Broekema et al. [17]. The program can set boundaries (constraints) for 36 nutrients and for energy to be fulfilled or limited through the upper boundaries (Table 1). In all scenarios explored in this study, the recommended daily intake (RDI) or adequate intake (AI) of nutrients was set as the lower boundaries, and tolerable upper intake level (UL) and maximum reference value (MRV) were set as the upper boundaries. The model combines the data from the Dutch National Food Survey and Food Composition Database and the Life Cycle Assessment databases, resulting in a consolidated dataset with daily amounts consumed, nutritional value, popularity estimates, and carbon footprint and estimates for food items.

For this purpose, a dataset of 207 food products, together representing the main foods consumed in the Netherlands, was used [14], which were distributed into 13 product categories. An overview of product categories is provided in Table 2. An overview of all products in each category is provided in Supplementary Table S1. For all food products, nutrient levels were derived from the Dutch Food Composition Database [19], whereas the environmental impact of data greenhouse gas emission and land use were derived as described by [14]. All data were thus representative for the Dutch situation. The nutrient levels in foods used are total levels and do not contain corrections for bioavailability. Price data were supermarket prices collected from the largest supermarket chain in the Netherlands.

### 2.2. Optimization Algorithm

The goal of the optimization is to find a diet as similar as possible to the reference diet while satisfying the set of optimization constraints. Similarity is determined by a deviation function

$$deviation=\sum_{i=1}^{207}{\left(x_{i}^{*}-x_{i}\right)}^{2}$$

where *i* indicates each of the food items available (i.e., 207 in total), $x_{i}$ is the value, in grams, of product *i* in the reference diet, and $x_{i}^{*}$ is the value, in grams, of the same product *i* in the optimized diet. The optimization diet is found by minimizing the deviation function subject to all constraints. In words, the deviation function sums up the square change, in grams, of the consumption of each food item available, and the optimization goal is to minimize the deviation. The practical effect of a quadratic function is that changes tend to be spread toward multiple products instead of concentrated in a few products.

### 2.3. Scenario Exploration

The starting point for our studies was an average Dutch diet according to the Dutch Food Consumption Survey [14,17], which was optimized to ensure that levels for all nutrients included in the optimization were between lower and upper boundaries (Table 1). This nutrient adequate diet, further referred to at the original, was the starting point for exploration of further scenarios.

Scenarios explored in this study consisted of the setting of one specific product category at a set intake level (0–1000 g, at 20 g intervals), after which the model was allowed to optimize the diet to ensure that levels for all nutrients included in the optimization (Table 1) were equal to or above the lower boundary and equal to or below the upper boundary. Subsequently, the impact of such dietary changes on environmental impact, price, and dietary composition (on a product category level), as well as on levels of macronutrients and micronutrients in optimized diets, were assessed. The following aspects were considered:

- Carbon footprint, land, price, amount of different food groups, and concentrations of protein, fat, and carbohydrate in the calculated diet were expressed as a % of the value for original diet (see above);
- Micronutrient levels, i.e., of minerals and vitamins, were expressed as a % of the RDI of each nutrient, with the particular aim of identifying which nutrients are likely to have the most impact on diet optimization.

## 3. Results

### 3.1. Impact of Dietary Shifts on Climate Impact and Price

**Figure 1:**
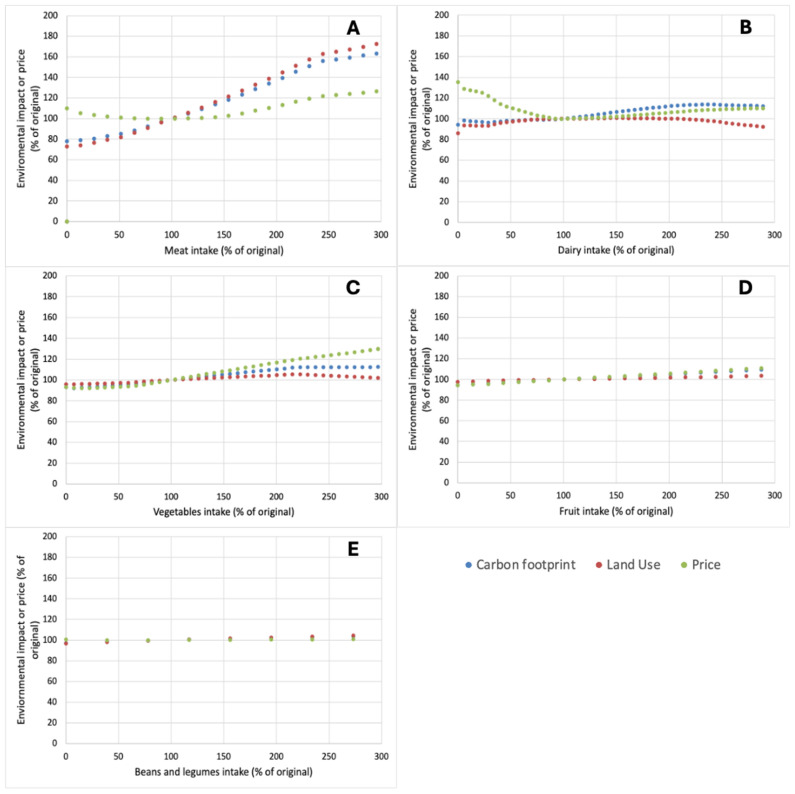
Impact of changes in the amount of meat (**A**), dairy (**B**), vegetables (**C**), fruit (**D**), or beans and pulses (**E**) on the carbon footprint, land use, and price of optimized nutrient-adequate diets. Both changes in food groups and outcomes are expressed as a percentage of levels in the original diet.

In this study, scenarios for the impact of shifts in product categories on the global warming potential, land use, and price of data in a Dutch context were explored, i.e., starting with the base diet: the average Dutch diet according to the Dutch Food Consumption Survey (RIVM). For this, we selected five different food groups for which shifts were imposed, i.e., meat, dairy, vegetables, fruits, and beans and pulses. As outlined in Figure 1, shifts in some food groups had a notably different effect on the impact of diets, whereas shifts in other food groups had little or no effect. Data in Figure 1 and subsequently figures are expressed as a percentage shift in the amount of a specific food group in the original diet. This enables better assessment of the proportional dietary shifts rather than weight-based ones. Within this context, the range of shifts from 0 to 300% was considered, i.e., from complete removal of the food group to a three-fold increase therein.

In terms of carbon footprint and land use, the largest effects were observed with changes in meat consumption. Reducing meat intake leads to a reduction in carbon footprint and land use, both by ~25% if no meat products are included in the diet (Figure 1A). Considering that meat contributed ~36% of the carbon footprint of the base diet, it suggests that products included in the diet to compensate for any nutrient gaps arising from removal of meat from the base diet have a notably lower carbon footprint than the meat products that were present in the base diet. The meat consumption above the levels of the base diet increased both the carbon footprint and land use of the diet (Figure 1A). After meat, dairy products were the largest contributor to the carbon footprint of the base diet, with ~16% of total carbon footprint of the base diet attributable to dairy products. However, as outlined in Figure 1B, diets with an enforced lower amount of dairy products were not notably lower in carbon footprint, nor in land use. For both, carbon footprint and land use, even complete removal of dairy from the diet caused <5% change compared to the base diet (Figure 1B). This suggests that the changes in diet composition required to fill the nutrient gaps arising from the removal of dairy products constitute a similar carbon footprint and land use; in other words, there seems to be little or no net gain from the product category replacements. An increase in dairy intake, up to 300% of the current intake, caused moderate increases in both carbon footprint and land use (Figure 1B). For vegetables (Figure 1C), fruit (Figure 1D), and beans and pulses (Figure 1E), only a limited effect of reducing these product categories did not majorly impact the overall carbon footprint and land use of the diets. In the base diet, vegetables, fruit, and beans and pulses contributed ~9, 4, and 2% of the total carbon footprint. Hence, particularly for fruit and beans and pulses, their removal alone would not be expected to notably remove the carbon footprint of the diet. Also, the dietary changes required to address nutrient gaps arising from these changes had little overall impact on carbon footprint. Increasing fruits (Figure 1D) and beans and pulses (Figure 1E) impacted the carbon footprint and land use of the diets only to a limited extent, whereas both parameters increased somewhat with increasing vegetable intake (Figure 1C); at 300% intake compared to the base diet, carbon footprint had increased by ~15%.

In addition to carbon footprint and land use, the impact of dietary changes on price was also evaluated. From Figure 1, it is clear that none of the scenarios evaluated caused a substantial reduction in price. The only slight reductions in price were observed in decreasing the levels of vegetables (Figure 1C) and fruit (Figure 1D) in the diets. Changes in levels of beans and legumes did not affect the price at all (Figure 1E) whereas for meat (Figure 1A) and particularly for dairy (Figure 1B), the base diet showed the lowest price, and both decreases and increases the levels of these product categories resulted in the increased price of the diet. For dairy products, Figure 1B shows that their omission from the diets resulted in a diet that was >35% more expensive. This can be related to the fact that the products required to replace the nutrient gaps arising from the removal of dairy are more expensive.

### 3.2. Impact of Dietary Shifts on the Food Group Composition of Diets

**Figure 2:**
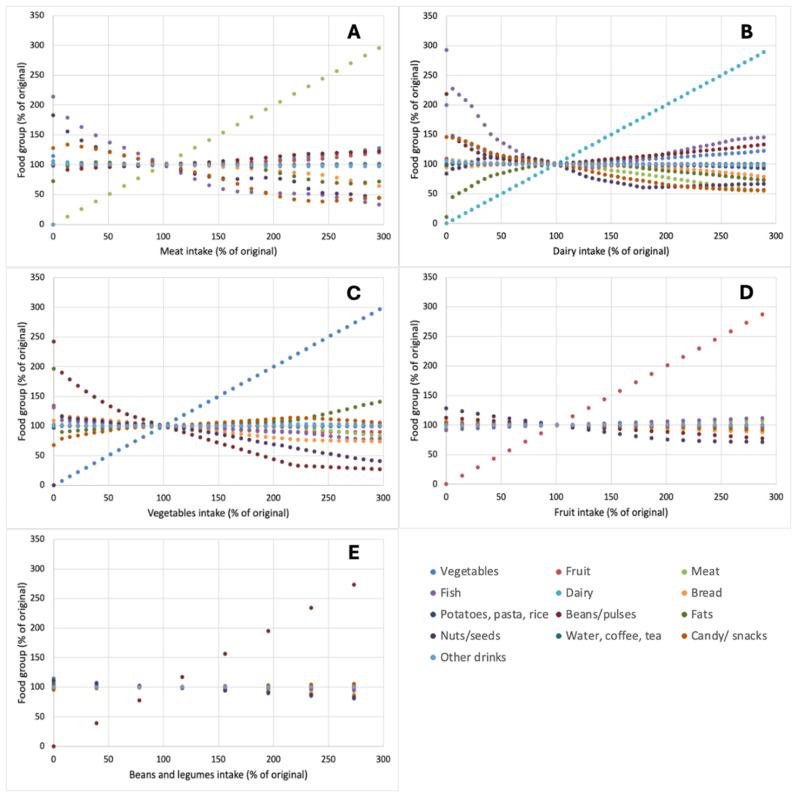
Impact of changes in the amount of meat (**A**), dairy (**B**), vegetables (**C**), fruit (**D**), or beans and pulses (**E**) on the amount to which different food groups are included in optimized nutrient-adequate diets. Both changes in food groups and outcomes are expressed as a percentage of levels in the original diet.

In addition to changes in climate impact metrics and price, the dietary composition of the optimized diets for the investigated scenarios was also investigated and is shown in Figure 2. In these figures, like in Figure 1, the base diet represents 100% of the respective product categories and changes were examined as a percentage thereof, i.e., between 0 and 300% of the base diet. As shown in Figure 2A, the product categories most affected by inducing constraints in the amount of meat in the diet were fish, nuts and seeds, and candy and snacks, whose contents were all inversely proportional to the number of meat products in the diet (Figure 2A). The categories fats and bread also decreased with increasing amounts of meat in the diet but were not impacted by decreasing levels of meat in the diet. This suggests that these product categories primarily contribute to the diet with components that are less required when meat intake increases but cannot compensate for deficiencies when the amount of meat in the diet is reduced. This could be primarily a caloric swap between meat and bread and fats.

When amounts of dairy in the diet were reduced, increases in fish and candy and snacks were also observed, but next to those, there were also strong increases in vegetables and beans and pulses (Figure 2B). The latter two categories were notably less affected by reductions in meat in the diet but are likely required to compensate for nutrient deficiencies arising from the removal of dairy. These would include, e.g., calcium and protein. Interestingly, amounts of the category fats in the diet also strongly decreased with dairy decreased (Figure 2B). For vegetables, the most notable changes were observed in beans and pulses and nuts and seeds when constraints on the number of vegetables were set (Figure 2C); whereas, constraints on fruits (Figure 2D) and beans and pulses (Figure 2E) had a limited impact on other food categories, presumably because of their limited contribution to overall nutrient intake in the base diet.

### 3.3. Impact of Dietary Shifts on the Macro- and Micronutrient Composition of Diets

**Figure 3:**
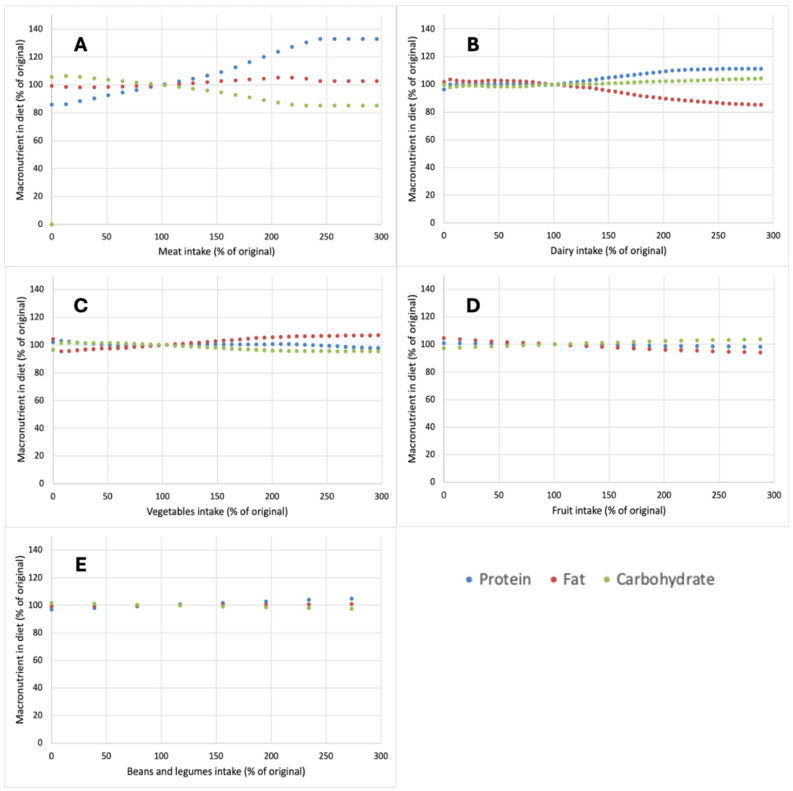
Impact of changes in the amount of meat (**A**), dairy (**B**), vegetables (**C**), fruit (**D**), or beans and pulses (**E**) on the amount to which the macronutrients protein, fat, and carbohydrate are included in optimized nutrient adequate diets. Both changes in food groups and outcomes are expressed as a percentage of levels in the original diet.

When considering macronutrient composition for the various optimized diets, it was noted that for both increases and decreases in vegetables (Figure 3C), fruits (Figure 3D), and beans and legumes (Figure 3E) or for decreases in dairy (Figure 3B), no impact on the levels of fat, protein, and carbohydrate compared to the base diet was observed. For increases in dairy, the optimized diets were lower in fat and higher in protein and also slightly higher in carbohydrate (Figure 3B). The constraints in levels of meat caused the biggest shifts in macronutrient levels in the optimized diets (Figure 3A). With increasing levels of meat, the protein increased, and the carbohydrate content decreased, whereas fat remained rather constant (Figure 3A).

**Figure 4:**
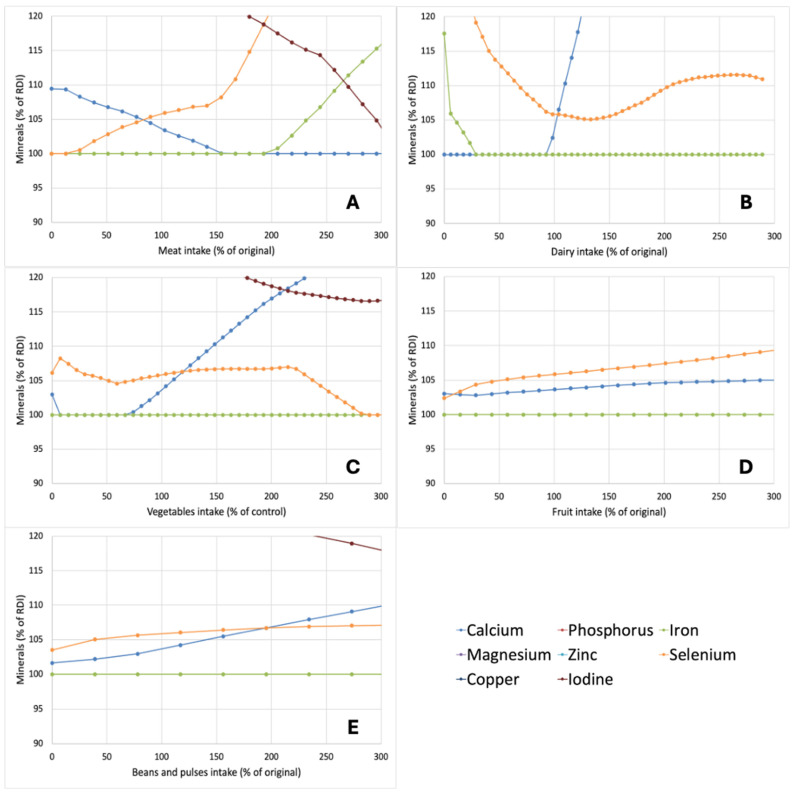
Impact of changes in the amount of meat (**A**), dairy (**B**), vegetables (**C**), fruit (**D**), or beans and pulses (**E**) on the amount to which the minerals are included in optimized nutrient-adequate diets. Changes in food groups and outcomes are expressed as a percentage of levels in the original diet whereas mineral levels are expressed as a percentage of the RDI of the minerals. Minerals not visible in graphs were at >120% at all datapoints.

**Figure 5:**
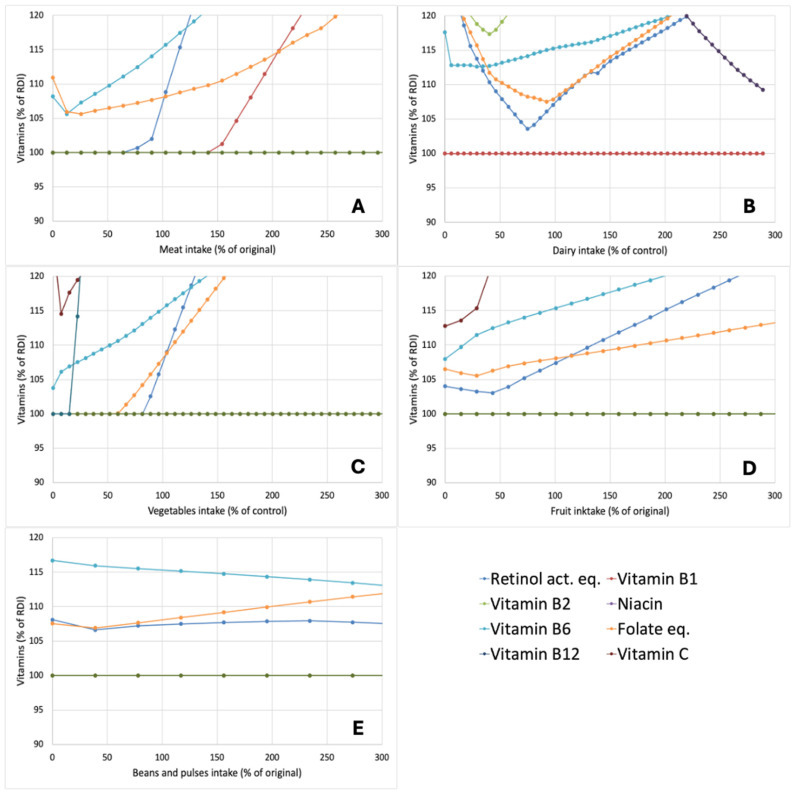
Impact of changes in the amount of meat (**A**), dairy (**B**), vegetables (**C**), fruit (**D**), or beans and pulses (**E**) on the amount to which the vitamins are included in optimized nutrient-adequate diets. Changes in food groups and outcomes are expressed as a percentage of levels in the original diet whereas mineral levels are expressed as a percentage of the RDI of the vitamins. Vitamins not visible in graphs were at >120% at all datapoints.

In addition to the effects of variation in the aforementioned food groups on the macronutrient composition of optimized diets, their effects on micronutrient levels were studied as well. For this purpose, the levels of minerals (Figure 4) and vitamins (Figure 5) in the diets were expressed as a percentage of the RDI value. As many micronutrients were present in considerable excess in the optimized diets, only those between 100 and 120% of the RDI values are shown in Figure 4 and Figure 5. For the minerals (Figure 4), four of the minerals were found to be at or close to the RDI in different dietary contexts, i.e., calcium, iron, selenium, and iodine. The other minerals (phosphorus, zinc, magnesium, and copper) were always present at levels >120% of the RDI in optimized diets. Particularly, iron was always close to or at the RDI in optimized diets (Figure 4). Reducing meat intake also brought selenium values closer to the RDI (Figure 4A), whereas, as expected, reducing the dairy intake brought calcium levels to the RDI (Figure 4B), indicating that meeting calcium requirements is a key challenge in creating nutrient-adequate diets with reduced dairy intake. Similar trends for calcium were also observed when vegetable intake was reduced (Figure 4C), which is in line with the fact that next to dairy, vegetables are a major contributor to Ca intake in the diet. For shifts in fruit intake (Figure 4D) and beans and pulses intake (Figure 4E), the shifts were proportionally smaller. This is in line with their overall smaller contributions to nutrient intake in the diets. For vitamins, several vitamins were always at or close to the RDI. Both vitamin B1 and vitamin D in pretty much all scenarios were at the RDI (Figure 5), indicating their large role in diet optimization. Of the vitamins, only vitamin C was present at levels >120% in almost all cases.

## 4. Discussion

### 4.1. Impact of Dietary Shifts on Diet Composition and Impact

In this study, we evaluated the impact of set amounts of food product categories (e.g., dairy, meat, vegetables, fruit, beans and pulses, etc.) on the composition as well as environmental impact and price of diets optimized for nutritional adequacy with minimum change from existing diets, i.e., we assessed the impact of changes that consumers could implement today by increasing or decreasing levels of specific food groups in their current diet, rather than designing future diets, which would require extensive changes to the food system and could take very long to enable implementation. Research shows that the majority of consumers are unlikely to change their entire diet at once but are more likely to increase or decrease the consumption of certain food items if they believe this will provide a benefit, e.g., in terms of personal health or environmental health [20,21]. In this respect, often, suggested changes include the reduced consumption of meat or dairy and the increased consumption of vegetables, fruit, or beans and legumes [1]. However, when the consumption of certain foods or food groups in the diet is reduced, their nutrients may need to be replaced by the inclusion of other sources. For instance, with approx. 60% of the total Ca intake in the Dutch diet coming from dairy products [22] and Ca intake barely meeting the requirements [13,23,24], the replacement of dairy is not easy. Hence, the assessment of the impact on diet level is critical, which is often ignored in product-based approaches such as nutritional LCAs.

As is clear from Figure 1, the only product category for which a reduction resulted in a notable reduction in environmental impact was meat. This could be explained on the basis of the sizeable contribution of meat to the environmental impact of the base diet but also based on the fact that although meat can contribute to many essential nutrients, several of them were present in considerable excess in the starting diet, which was created based on the Dutch food consumption survey [22]. For instance, although meat is an excellent source of high-quality protein [25,26], protein levels in the Dutch diet are considerably higher than the RDI for protein [22,27]. Hence, the protein that is removed from the diet as a result of reduced meat intake does not need to be replaced. Another sizeable contributor to protein intake in the starting diet was the category of dairy products. However, their reduction in the diet did not notably impact climate impact (Figure 1), in line with previous observations [14]. This can be attributed to the fact that dairy products contribute many essential nutrients to the diet [28] and their replacement also comes with a sizeable environmental impact.

A further consideration is whether calculated alternative diets are acceptable to consumers. While the calculated nutrient-adequate diets with reduced meat intake indeed did have a lower environmental impact (Figure 1), such diets also showed notable increases in, e.g., vegetables, which approximately doubled (Figure 2). For the removal of dairy, even larger increases in required vegetable intake to attain nutrient adequacy were found (Figure 2). These also led to an increase in price (Figure 1), which will not be feasible for all consumers, nor may such large increases in vegetable intake fit in current eating habits.

### 4.2. Sustainable Diets: Focus Beyond Protein

Although proposed dietary shifts with the aim of reducing the environmental impact of diets are often placed in the context of the so-called protein transition [29,30,31], the analysis of optimized nutritional profiles did not highlight protein as a limiting nutrient. In all cases, the calculated protein intake was above the RDI (Figure 3) and it was actually the micronutrients, most notably the minerals iron, calcium, selenium, and iodine, as well as vitamin A and vitamin B12, which were close to RDI and thus considered as the main nutrients ‘driving’ the model (Figure 4 and Figure 5). As such, while the term protein transition may appeal from an aspirational perspective, it can be considered risky in drawing particular attention to a nutrient that is actually not limiting, at least not in diets in Western countries [32], where the ‘protein transition’ appears to gain most momentum. The aforementioned micronutrients are derived primarily from animal-based foods and in scenarios where animal-based products were strongly reduced, there was an inherent risk of limitation [33]. For instance, 55–60% of Dutch dietary intake is from dairy products and their removal from diets would necessitate the increased consumption of other calcium sources or the inclusion of new calcium sources. The outcomes of the optimizations performed in this work suggest that the replacements required, e.g., in the form of a strongly increased intake of vegetables, do not notably reduce GWP or land use while increasing the price of the overall diet (Figure 1).

## 5. Conclusions

In conclusion, changes toward diets with a lower environmental footprint should be achievable and affordable for consumers. In this study, we have shown for the Dutch context that a sustainable diet is mostly plant-based and achieves nutrient adequacy optimized with animal-based products. The outcomes of this work clearly highlight the importance of considering the shift toward more sustainable food systems on a diet basis, rather than on a product or food group basis, as is often done. Because diets are required to be nutrient-adequate, the removal of products with a perceived high environmental impact does not necessarily reduce the impact of the whole diets as replacements to retain nutrient adequacy will also have an impact. Hence, diet-based approaches are critical. From the current study, it would appear that while a reduction in meat products can indeed reduce the climate impact of the diets, that of dairy products is unlikely to do so. Hence, while sustainable diets will include a shift to plant-based, they will still require animal-based products as critical sources of nutrients for optimal nutrient-adequate diets.

## Figures and Tables

**Table 1:** Lower and upper boundaries for macronutrients, vitamins, and minerals including in dietary optimization.

Property	Property Group	Lower Boundary	Upper Boundary
Energy (kcal)	Macronutrients	2000	2000
Protein total (g)	Macronutrients	50	125
Fat total (g)	Macronutrients	44.4	88.9
SAFA (g)	Macronutrients	0	22.2
PUFA (g)	Macronutrients	0	26.7
Linoleic acid (g)	Macronutrients	4.44	1,000,000
ALA (g)	Macronutrients	2.22	1,000,000
Trans fatty acids (g)	Macronutrients	0	2.22
Cholesterol (mg)	Macronutrients	0	300
Carbohydrates total (g)	Macronutrients	200	350
Fiber (g)	Macronutrients	30	1,000,000
Water (g)	Macronutrients	2300	3800
Alcohol (g)	Macronutrients	0	10
DHA + EPA (mg)	Macronutrients	450	1,000,000
Retinol eq. (μg)	Vitamins	0	1,000,000
Retinol act. eq. (μg)	Vitamins	700	3000
Vitamin B1 (mg)	Vitamins	1.1	1,000,000
Vitamin B2 (mg)	Vitamins	1.1	1,000,000
Niacin (mg)	Vitamins	13	1,000,000
Vitamin B6 (mg)	Vitamins	1.5	25
Folate eq. (μg)	Vitamins	300	1000
Vitamin B12 (μg)	Vitamins	2.8	1,000,000
Vitamin C (mg)	Vitamins	75	1,000,000
Vitamin D (μg)	Vitamins	3.3	100
Vitamin E (mg)	Vitamins	8	300
Vitamin K total (ug)	Vitamins	90	1,000,000
Vitamin K2 (ug)	Vitamins	0	1,000,000
Calcium (mg)	Minerals	1000	2500
Phosphorus (mg)	Minerals	600	3000
Iron (mg)	Minerals	15	25
Sodium (mg)	Minerals	0	1,000,000
Potassium (mg)	Minerals	3100	2400
Magnesium (mg)	Minerals	280	1,000,000
Zinc (mg)	Minerals	7	530
Selenium (μg)	Minerals	50	25
Copper (mg)	Minerals	0.9	300
Iodine (μg)	Minerals	150	5

**Table 2:** Food product categories used in this research.

Product Categories	
Vegetables	Beans and pulses
Fruit	Fats
Meat	Nuts and seeds
Fish	Water, coffee tea
Dairy	Candy and snacks
Bread	Other drinks
Potatoes, pasta, rice	

## Data Availability

Dataset available on request from the authors. The data are not publicly available due to.

## Supplementary Materials

The following supporting information can be downloaded at https://www.mdpi.com/article/10.3390/nu17020343/s1: Table S1: List of products and NEVO code for each product.

## References

[1] Willett W., Rockström J., Loken B., Springmann M., Lang T., Vermeulen S., Garnett T., Tilman D., DeClerck F., Wood A. (2019). Food in the Anthropocene: The EAT–Lancet Commission on Healthy Diets from Sustainable Food Systems. Lancet.

[2] Poore J., Nemecek T. (2018). Reducing food’s environmental impacts through producers and consumers. Science.

[3] Smith N.W., Fletcher A.J., Hill J.P., McNabb W.C. (2021). Animal and plant-sourced nutrition: Complementary not competitive. Anim. Prod. Sci..

[4] Smith N.W., Fletcher A.J., Hill J.P., McNabb W.C. (2022). Modeling the contribution of milk to global nutrition. Front. Nutr..

[5] Smith N.W., Fletcher A.J., Hill J.P., McNabb W.C. (2022). Modeling the contribution of meat to global nutrient availability. Front. Nutr..

[6] Afshin A., Sur P.J., Fay K.A., Cornaby L., Ferrara G., Salama J.S., Mullany E.C., Abate Z., Afarideh M. (2019). Health Effects of Dietary Risks in 195 Countries, 1990–2017: A Systematic Analysis for the Global Burden of Disease Study 2017. Lancet.

[7] Nemecek T., Jungbluth N., Mila i Canals L.M., Schenck R. (2016). Environmental impacts of food consumption and nutrition: Where are we and what is next?. Int. J. Life Cycle Assess..

[8] Notarnicola B., Sala S., Anton A., McLaren S.J., Saouter E., Sonesson U. (2017). The role of life cycle assessment in supporting sustainable agri-food systems: A review of the challenges. J. Clean. Prod..

[9] McLaren S., Berardy A., Henderson A., Holden N., Huppertz T., Jolliet O., De Camillis C., Renouf M., Rugani B., Saarinen M. (2021). Integration of Environment and Nutrition in Life Cycle Assessment of Food Items: Opportunities and Challenges.

[10] Bianchi M., Strid A., Winkvist A., Lindroos A.K., Sonesson U., Hallström E. (2020). Systematic evaluation of nutrition indicators for use within food LCA studies. Sustainability.

[11] McAuliffe G.A., Takahashi T., Lee M.R. (2020). Applications of nutritional functional units in commodity-level life cycle assessment (LCA) of agri-food systems. Int. J. Life Cycle Assess..

[12] McAuliffe G.A., Takahashi T., Beal T., Huppertz T., Leroy F., Buttriss J., Collins A.L., Drewnowski A., McLaren S.J., Ortenzi F. (2023). Protein quality as a complementary functional unit in life cycle assessment (LCA). Int. J. Life Cycle Assess..

[13] Shkembi B., Huppertz T. (2021). Calcium absorption from food products: Food matrix effects. Nutrients.

[14] Kramer G.F., Tyszler M., van’t Veer P., Blonk H. (2017). Decreasing the overall environmental impact of the Dutch diet: How to find healthy and sustainable diets with limited changes. Public Health Nutr..

[15] Van Dooren C., Aiking H. (2016). Defining a nutritionally healthy, environmentally friendly, and culturally acceptable Low Lands Diet. Int. J. Life Cycle Assess..

[16] Van Dooren C., Tyszler M., Kramer G.F., Aiking H. (2015). Combining low price, low climate impact and high nutritional value in one shopping basket through diet optimization by linear programming. Sustainability.

[17] Broekema R., Tyszler M., van’t Veer P., Kok F.J., Martin A., Lluch A., Blonk H.T. (2020). Future-proof and sustainable healthy diets based on current eating patterns in the Netherlands. Am. J. Clin. Nutr..

[18] Bruins M.J., Létinois U. (2021). Adequate vitamin D intake cannot be achieved within carbon emission limits unless food is fortified: A simulation study. Nutrients.

[19] NEVO: Dutch Food Composition Database. https://nevo-online.rivm.nl.

[20] Payró C., Taherzadeh O., van Oorschot M., Koch J., Marselis S. (2024). Consumer resistance diminishes environmental gains of dietary change. Environ. Res. Lett..

[21] Röös E., Tjärnemo H. (2011). Challenges of carbon labelling of food products: A consumer research perspective. Br. Food J..

[22] van Rossum C.T.M., Buurma-Rethans E.J.M., Dinnissen C.S., Beukers M.H., Brants H.A.M., Dekkers A.L.M., Ocké M.C. (2020). The Diet of the Dutch: Results of the Dutch National Food Consumption Survey 2012–2016.

[23] Miller G.D., Jarvis J.K., McBean L.D. (2001). The importance of meeting calcium needs with foods. J. Am. Coll. Nutr..

[24] Smith N.W., Fletcher A.J., Dave L.A., Hill J.P., McNabb W.C. (2021). Use of the DELTA model to understand the food system and global nutrition. J. Nutr..

[25] Hodgkinson S.M., Montoya C.A., Scholten P.T., Rutherfurd S.M., Moughan P.J. (2018). Cooking conditions affect the true ileal digestible amino acid content and digestible indispensable amino acid score (DIAAS) of bovine meat as determined in pigs. J. Nutr..

[26] Adhikari S., Schop M., de Boer I.J., Huppertz T. (2022). Protein quality in perspective: A review of protein quality metrics and their applications. Nutrients.

[27] Dinnissen C.S., Ocké M.C., Buurma-Rethans E.J., van Rossum C.T. (2021). Dietary changes among adults in The Netherlands in the period 2007–2010 and 2012–2016. Results from two cross-sectional national food consumption surveys. Nutrients.

[28] Sanderman-Nawijn E.L., Brants H.A.M., Dinnissen C.S., Ocké M.C., van Rossum C.T.M. (2024). Energy and Nutrient Intake in The Netherlands. Results of the Dutch National Food Consumption Survey 2019–2021.

[29] Aiking H., de Boer J. (2020). The next protein transition. Trends Food Sci. Technol..

[30] Hundscheid L., Wurzinger M., Gühnemann A., Melcher A.H., Stern T. (2022). Rethinking meat consumption–How institutional shifts affect the sustainable protein transition. Sustain. Prod. Consum..

[31] Jenkins W.M., Trindade L.M., Pyett S., van Mierlo B., Welch D., van Zanten H.H. (2024). Will the protein transition lead to sustainable food systems?. Glob. Food Secur..

[32] Moughan P.J. (2021). Population protein intakes and food sustainability indices: The metrics matter. Glob. Food Secur..

[33] Beal T., Gardner C.D., Herrero M., Iannotti L.L., Merbold L., Nordhagen S., Mottet A. (2023). Friend or foe? The role of animal-source foods in healthy and environmentally sustainable diets. J. Nutr..

