# Supplementary material for: Exploring Nutrient-Adequate Sustainable Diet Scenarios That Are Plant-Based but Animal-Optimized
Source: Nutrients. 2025 Jan 18;17(2):343. doi: 10.3390/nu17020343 (PMC11767869; doi:10.3390/nu17020343)
Supplement: Supplementary file 1 [file nutrients-17-00343-s001.zip › nutrients-3428623-supplementary.pdf]

Supplementary Table S1: List of products and NEVO code for each product

NEVO	Product name
7	Endive raw
8	Endive boiled
15	Cauliflower boiled
16	Kale curly boiled
20	Mushrooms boiled
26	Celeriac boiled
32	Sweet pepper green boiled
37	Leek boiled
54	Cabbage oxheart boiled
55	Brussel sprouts boiled
59	Bean sprouts boiled
63	Onions raw
64	Onions boiled
68	Chicory boiled
71	Carrots raw average
72	Carrots boiled average
74	Cabbage sauerkraut cooked
84	Eggs chicken boiled average
111	Mussels boiled
122	Crisps potato average
136	Peas and carrots tinned
137	Olives tinned/glass
141	Tomato puree concentrated tinned
146	Spinach frozen boiled
148	Strawberries
150	Pineapple
151	Banana
160	Grapes with skin average
165	Manderins
166	Melon netted
171	Orange
179	Apple sauce tinned
199	Cashew nuts unsalted
204	Peanuts unsalted
206	Walnuts unsalted
207	Nuts mixed unsalted
227	Crispbakes Dutch
229	Crispbread averaged
230	Roll white soft
236	Bread brown wheat

240	Cake Dutch spiced Ontbijtkoek
242	Bread rye dark
246	Bread wholemeal average
248	Bread white water based
251	Apple pie Dutch w shortbread w marg
252	Biscuit sweet
253	Cake wo butter
254	Cake sponge Dutch Eierkoek
257	Almond paste filled tarts average
258	Biscuits averaged
261	Biscuit spiced Speculaas
263	Biscuit brown/wholemeal
266	Snack sausage roll puff pastry
272	Milk chocolate-flavoured full fat
276	Custard chocolate full fat
278	Yoghurt full fat
279	Milk whole
280	Coffee creamer full fat
282	Custard vanilla full fat
284	Yoghurt low fat with fruit
285	Coffee creamer half fat
286	Milk semi-skimmed
289	Buttermilk
294	Milk skimmed
301	Yoghurt low fat
303	Ice cream dairy cream based
310	Butter unsalted
313	Oil soy
317	Oil sunflower seed
322	Sausage Dutch Frikandel deep-fried
324	Sausage smoked cooked average
326	Croquette meat ragout deep-fried
335	Liver pate
340	Beef smoke-dried
348	Shrimps Dutch peeled boiled
350	Herring salted
377	Sugar granulated
383	Juice apple
390	Beer pilsner
395	Soft drink cola with caffeine
400	Soft drink wo caffeine
410	Juice orange pasteurized
417	Juice drink

421	Whisky
422	Wine red
423	Wine white dry
427	Syrup apple
431	Chocolate milk
432	Chocolate plain
436	Spread chocolate hazelnut
443	Honey
445	Jam
451	Mayonnaise
455	Peanut butter
462	Ketchup tomato
463	Fruit drink concentrate undiluted
465	Sauce for chips 25% oil
467	Pancake
497	Fruit drink concentrate Roosvicee Origin
511	Cheese Edam 40+
513	Cheese Gouda 48+ average
515	Cheese spread 48+
562	Leek raw
567	Sausage luncheon meat
584	Ketchup curry
601	Oil olive
604	Mackerel steamed
616	Peanut sauce jar prepared
638	Salami sausage saveloy
639	Bacon rashers streaky
644	Coffee prepared
645	Tea prepared
655	Crispbakes Dutch wholemeal
657	Yoghurt drink
658	Rice white boiled
659	Pasta white average boiled
660	Beans brown tinned
713	Waffle syrup average
747	Mineral water average
761	Soup clear with meat and vegetables
762	Soup clear w meat vegetables and noodles
766	Soup main course w legumes and meat
784	Ham lean boiled
785	Ham shoulder medium fat boiled
797	Soup vegetable based dried packet prep
807	Jam without sugar

810	Beef steak tartare spiced filet americ
818	White fish fillet in batter deep-fried
819	Cod boiled
863	Yoghurt full fat with fruit
875	Apple with skin average
879	Butter salted
884	Sweet pepper red raw
885	Sweet pepper red boiled
917	Fromage frais half fat w fruit
920	Broccoli boiled
951	Beans French boiled
953	Peas frozen boiled
958	Beetroot boiled
966	Courgettes boiled
969	Peas marrowfat legumes boiled
982	Potatoes wo skins boiled average
1014	Rice brown boiled
1056	Kiwi fruit
1095	Peas chick boiled
1096	Salmon smoked
1152	Salami
1162	Sausage cooked
1239	Liver pate sausage
1311	Chocolate confetti averaged
1326	Minced beef/pork shallow fried
1382	Cheese 30+ average
1389	Liquorice Dutch type average
1392	Chicken fillet prepared
1464	Milk chocolate-flavoured semi-skimmed
1468	Beer >7 vol% alcohol
1474	Ice lolly/ Sorbet
1502	Yoghurt half fat
1508	Chocolates filled/Belguim chocolate
1511	Hamburger vegetarian unprep
1521	Juice drink light
1522	Soft drink light without caffeine
1523	Cola light soft drink with caffeine
1524	Sauce tomato ready made jar
1528	Stock from cube prepared
1530	Butter product half fat
1536	Beef rump steak prepared
1539	Beef frying steak prepared
1540	Minced beef shallow fried

1551	Sausage pork Braadworst prepared
1556	Pork tenderloin prepared
1559	Pork shoulder chop prepared
1589	Tuna in oil tinned
1602	Drink soya several flavours Alpro
1610	Salmon farmed prep in microwave oven
1633	Chicken with skin prepared
1720	Custard several flavours full fat
1723	Cheese 20+
1885	Water average
2047	Mincemeat vegetarian unprepared
2059	Low fat margarine 40% fat <17 g sat
2062	Margarine 80% fat 17-24 g saturates
2066	Cooking fat liquid 97% fat <17 g sat
2072	Margarine product 60% fat <17 g sat
2077	Margarine liq 80% fat <17 g saturates
2081	Breakfast cereal Cornflakes
2086	Ice tea
2232	Biscuit fruit
2346	Lettuce average raw
2366	Muesli crunchy plain/w fruit
2405	Chocolate chip cookie
2444	Tea herbal instant sw prepared
2476	Cappuccino freshly made
2558	Margarine liq 80% fat < 17g sat unsalted
2654	Sandwich meat chicken
2657	Spread duo w chocolate
2734	Tomato average raw
2735	Tomato average boiled
2736	Rocket raw
2739	Cucumber w skin raw
2743	Sweet pepper average boiled
2748	Pear with skin
2755	Juice orange freshly squeezed
2765	Pangasius prep in microwave oven
2796	Roll brown hard
2803	Bun currant/raisin
2858	Drink soya Fresh Light Alpro
